# Wood’s Therapeutics and Mat. Med

**Published:** 1874-05

**Authors:** 


### Bibliographical.

A Treatise on Therapeutics, Comprising Materia Medica and Toxicology, *with especial reference to the application of the Physiological Action of Drugs to Clinical Medicine*, by H. C. Wood, Jr., M.D., Professor of Botany and Clinical Lecturer on Diseases of the Nervous System in the Medical Department of the University of Pennsylvania; Physician to the Philadelphia Hospital, etc., etc. Philadelphia: J. B. Lippincott & Co., 1874. octavo, cloth, pp. 578. Price $5.50. Sold in Atlanta by Phillips & Crew.

This is an entirely new work, and treating as it does the much hackneyed themes of Materia Medica and Therapeutics, the author feels an apology from him is demanded in bringing it before the public. Sufficient ground for this apology he finds in his own individual wants. "There are a number of excellent treatises upon Materia Medica and Therapentics, yet in various attempts at original research, as well as in the ward and lecture-room of the hospital, I have keenly felt the want of something more. There are many points of view from which a subject can be looked at; there are many paths by which it may be approached; and to me, other points of view, other modes of approach, have been far more enticing than those in our standard treatises."

As an instance of the peculiar views of the author, we quote from his preface: "In spite, then, of Dr. Niemeyer's assertion, that experiments made with medicaments upon the lower animals or upon healthy human beings have, as yet, been of no direct service to our means of treating disease, and that a continuation of such experiments gives no prospect of such service, it is certain that in these experiments is the only rational scientific ground-work for the treatment of disease. * * * It has been strenuously objected that drugs do not act upon the lower creatures in the same manner as they do upon man. When I first commenced the studies whose outcome is the present volume, I was profoundly impressed with the truth of this oft-repeated assertion, and with the difficulties which it put in the way. To-day I do not believe that, stated in its broad sense, it is true. Indeed, more strongly, I assert that it is not true," etc., etc.

Dr. Wood arranges remedies into two grand division, i. e., systemic and non-systemic. The former he divides into general and local; and general remedies are further subdivided into astringents, tonics, cardiac stimulants, cardiac sedatives, antispasmodics, analgesics, mydriatics, anæsthetics, excito-motors, depresso-motors, and alteratives. Under the head of local remedies he places emetics, cathartics, diuretics, diaphoretics, expectorants, emmenagogues, oxytocics, sialagogues, errhines, epispastics, rubefacients, escharotics, demulcents, emolients, diluents, and protectives. His sub-classes of non-systemic remedies include antacids, anthelmintics, digestants, absorbents, and disinfectants.

The pressure of other engagements prevents more than a hasty glance over the contents of the volume; our eye is arrested however by a few passages which we extract, as follows: "As an anæsthetic, ether does not act with the rapidity and pleasantness of choroform, but it has the advantage of safety. So dangerous is chloroform, and so safe is ether, that there is no excuse for the use of the former agent under ordinary circumstances. The reason of the safety of ether is that, unlike chloroform, it never suddenly paralyses the heart. It may kill by inducing asphyxia but it does so slowly, and in the great majority of cases after warning which can be overlooked only through the most reckless carelessness." In speaking of chloroform, he says: "It kills the robust, the weak, the well and the diseased alike, and the previous safe passage through one or more inhalations is no guarantee against its lethal action. Statistics seem to indicate a mortality of about one in three thousand inhalations; and hundreds of utterly unnecessary deaths have now been produced by the extraordinary persistence in its use by a portion of the profession. It ought, therefore, never to be employed except under especial circumstances, as in some cases of *puerperal eclampsia*, when a speedy action is desired, or in the field during war-time, when the bulkier anæsthetics cannot be transported."

In speaking of chloral, one of the depresso-motors, our author remarks: "the symptoms induced by chloral are only analogous to those caused by chloroform, *not identical*. * * * I think the evidence which has been adduced completely disproves the chloroform-theory, and forces assent to the proposition that chloral acts directly upon the organism. * * * The indication which it most usefully meets is *to induce sleep*.

The more purely nervous the wakefulness is, the more successful is the remedy. On the other hand, when severer pain causes wakefulness, chloral is of very little value, at least in doses which I think safe." No allusion is made to the exhibition of a moderate dose of chloral, supplemented by the inhalation of a few whiffs of chloroform to allay pain until the quiet sleep of the chloral supervenes. "In *whooping-cough* it would really seem to be of great value, etc. * * * That chloral is a dangerous agent, capable of destroying life, is attested by a number of published cases; but this is true of other drugs; and the practical point to be determined is, does it ever act out of proportion to the amount ingested? * * * Considerable attention has been given both in this country and abroad, to the subject of chronic chloral-poisoning; and, whilst some affections have been erroneously attributed to the drug, there seems to be no doubt that its long continued use, often does produce serious symptoms. * * * I think the practical deduction from these facts is that *twenty grains* is the highest safe dose of the remedy; that this amount should not be repeated oftener than once an hour, and, after sixty grains have been taken, not for some hours, unless in very urgent cases, as acute tetanus or violent chorea threatening speedy dissolution."

"The dose of *santonin* for an adult is two to four grains; for a child two years old, one-fourth to one-half grain. Very alarming symptoms have been caused by two one-grain doses exhibited within three hours in a child eight years old; in a child two and a half years old, four grains apparently came very near causing death; and in the fatal case noted, only two grains were taken by a child five years old. For young infants, santonin is hardly a safe remedy in any efficient dose. When a dose of any size is given, it should not be repeated in less than eight hours, and the last dose should be accompanied by a purgative amount of calomel." Our own more limited experience of the comparative safety of chloral and santonin does not accord with the observations of Dr. Wood.

Our author, in his work, is fully up with the times, and the publishers bring him before the reading public in a very handsome and attractive volume.

